# Effectiveness of Serial Membrane Sweeping and Stretching at Term for the Induction of Labour

**DOI:** 10.7759/cureus.101898

**Published:** 2026-01-20

**Authors:** Geyum Ete, Bindu Bajaj, Sheeba Marwah, Asmita Saran, Abhigya Malik, Nidhi Verma, Archana B.S.

**Affiliations:** 1 Obstetrics and Gynaecology, Vardhman Mahavir Medical College (VMMC) and Safdarjung Hospital, New Delhi, IND

**Keywords:** bishop score, cervical length, labour induction, membrane sweeping, term pregnancy

## Abstract

Objective: To evaluate the effectiveness of serial sweeping and stretching of the membrane at term for the induction of labour in low-risk pregnancies. Methods: This randomized controlled study was conducted over 18 months at Vardhman Mahavir Medical College (VMMC) and Safdarjung Hospital, from July 2023 to December 2024, and included 226 term pregnant women randomized into two groups: intervention (serial membrane sweeping) and control (no intervention). All women were followed for the onset of labour, need for formal induction, and mode of delivery. Data was analysed using IBM SPSS Statistics for Windows, Version 23 (Released 2016; IBM Corp., Armonk, New York, United States), with p < 0.05 considered statistically significant. Results: The intervention group demonstrated a significantly lower induction rate (29.2%) compared to the control (69.0%) (p < 0.001). Serial membrane sweeping was associated with a higher Bishop’s score at the second visit (6.58 vs. 4.63; p < 0.001) and shorter onset of labour (21.48 hours vs. 24.29 hours; p < 0.001). Correlation analysis revealed that higher Bishop scores and shorter cervical length significantly predicted earlier labour onset. Conclusion: Serial sweeping and stretching of membranes significantly reduced the need for formal induction and promoted earlier onset of labour, particularly in multigravida patients. The procedure is effective and safe in low-risk term pregnancies.

## Introduction

Approximately one-fourth of pregnant women require induction of labour for maternal or fetal indications. The most common indications include post-datism, hypertensive disorders of pregnancy, maternal medical comorbidities, fetal growth restriction, intrauterine fetal demise, and premature rupture of membranes [1,2].

Traditionally, labour induction has been performed using pharmacological or surgical methods such as artificial rupture of membranes, oxytocin infusion, or prostaglandin administration. However, sweeping and stretching of the membranes is a simple, non-invasive mechanical method that has gained renewed attention for cervical ripening at term [3].

The primary mechanism of this technique is to promote endogenous prostaglandin release by manually separating the chorioamniotic membranes from the decidua, thereby enhancing cervical favourability [4]. When the membranes are inaccessible, cervical stretching or massage may be performed to achieve a similar effect [5-7]. Common side effects include mild abdominal cramping, discomfort, spotting, light bleeding due to capillary disruption during cervical dilation, irregular contractions, and, rarely, leakage of amniotic fluid per vaginum.

Although the use of this traditional method declined with the introduction of pharmacological agents in recent decades, there has been a resurgence of interest in reintroducing membrane sweeping into modern obstetric practice. The World Health Organization now endorses this method as part of a positive pregnancy experience [8]. Membrane sweeping has been shown to reduce pregnancy duration by two to five days and to lower the need for formal induction (8.1% vs. 18.8% in case and control groups, respectively) [9].

Extensive research has examined the efficacy of membrane sweeping and stretching, both as standalone procedures and as adjuncts to formal induction. However, there remains no consensus regarding the optimal timing and frequency of membrane sweeping to prevent post-term pregnancies. Some studies have reported no significant reduction in the need for post-term induction with a single sweep, while others have demonstrated that serial sweeping and stretching are more effective in expediting delivery [10,11]. Given the limited literature on the efficacy of serial membrane sweeping and stretching for labour induction in the Indian population, the present study was conducted to evaluate this method as a safe and effective means of inducing labour at term in low-risk pregnancies in a tertiary care hospital setting.

## Materials and methods

**Figure 1:**
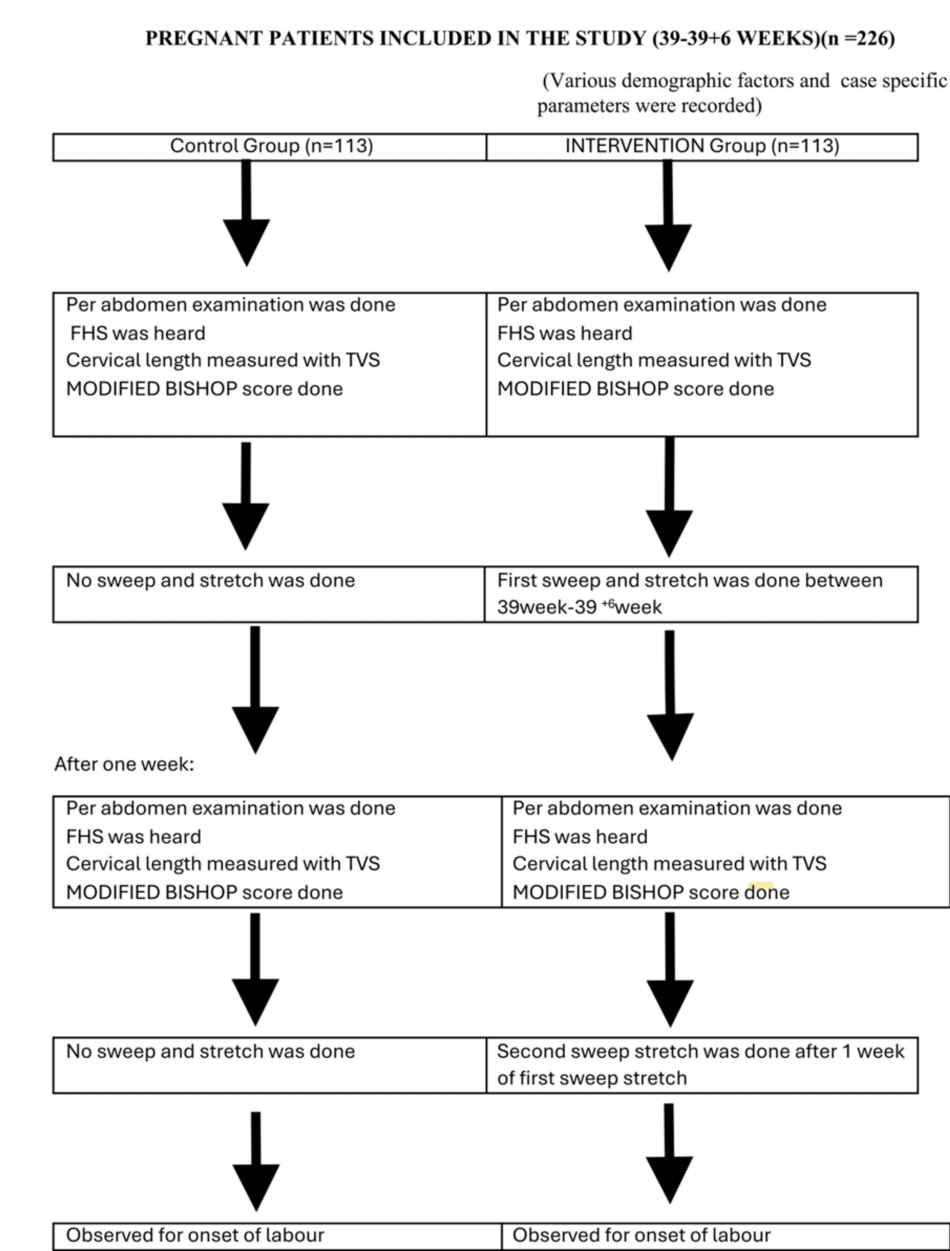
Flowchart depicting the study methodology n: number of participants in each group; TVS: transvaginal sonography; FHS: fetal heart sound

This was an open-label, randomized controlled study conducted in the Department of Obstetrics and Gynaecology, Vardhman Mahavir Medical College and Safdarjung Hospital, New Delhi, India, after obtaining approval from the Institutional Ethics Committee (approval no: IEC/VMMC/SJH/Thesis/06/2022/CC-151). The study was registered with the Clinical Trial Registry - India (ICMR-NIMS) under approval number CTRI/2023/10/059267. The study period extended from July 2023 to December 2024. All women with singleton pregnancies and cephalic presentations between 39 and 40 weeks of gestation who had no contraindication to vaginal delivery were included after providing written informed consent. High-risk pregnancies (e.g., placenta previa, previous cesarean section, malpresentation, multiple gestation, or medical contraindications to labour) were excluded. Detailed maternal history, demographic data, and obstetric examination findings were recorded. Participants were then randomized into two groups: the intervention group and the control group, using a computer-generated randomization sequence, with 113 participants in each arm. The Bishop’s score was assessed, and transvaginal sonography (TVS) was performed to measure cervical length in all participants. The intervention group (Group I) underwent serial sweeping and stretching of the membranes between 39 and 39+6 weeks, up to a maximum of two times, one week apart. The control group, on the other hand, did not undergo membrane sweeping or stretching. Participants were instructed to report to the hospital in case of pain, vaginal bleeding, or leakage of fluid. Those who went into spontaneous labour were followed through delivery and postpartum discharge. Women who did not go into labour were induced as per hospital protocol. The flowchart in Figure 1 depicts the study methodology.

Measurement of cervical length (TVS)

Participants were asked to empty the bladder before the examination. A longitudinal view of the cervix was obtained, clearly identifying the cervical canal and surrounding mucosa. Appropriate magnification was used, and minimal pressure was applied to the cervix with the probe. The duration of the examination was approximately three to five minutes.

Procedure of membrane sweeping

After ensuring an empty bladder, a per vaginal examination was performed under aseptic precautions. The modified Bishop’s score was assessed. If the pelvis was deemed adequate and no cephalopelvic disproportion was suspected, membrane sweeping was carried out by inserting the index finger through the cervix and rotating it circumferentially (360 degrees) against the uterine wall to separate the chorionic membranes from the decidua. Any occurrence of pain, vaginal bleeding, or rupture of membranes was recorded. If the cervix was closed during the initial attempt, the participant was re-evaluated after one week for a repeat procedure in the intervention group.

Sample size calculation

The sample size of 226 was determined based on the study by Saichandran et al. (2015), who reported the proportion of patients with spontaneous labor as 64% in Group I and 47% in Group II.

Outcomes measured

The primary outcome measured was the duration to onset of labour, and secondary outcomes were the need for further induction of labour and mode of delivery, besides foetal outcomes.

Statistical analysis

Data was recorded in an MS Excel spreadsheet program (Microsoft Corp., Redmond, WA, USA) and analyzed using IBM SPSS Statistics for Windows, Version 23 (Released 2016; IBM Corp., Armonk, New York, United States). Group comparisons for continuously distributed data were made using the independent sample t-test when comparing two groups, and one-way analysis of variance (ANOVA) when comparing more than two groups. Post-hoc pairwise analysis was performed using Tukey's honestly significant difference test (Tukey's HSD) in the case of one-way ANOVA to control for alpha inflation. If data were found to be non-normally distributed, appropriate non-parametric tests in the form of the Wilcoxon test/Kruskal-Wallis test were used for these comparisons.

Chi-square test was used for group comparisons for categorical data. In case the expected frequency in the contingency tables was found to be <5 for >25% of the cells, Fisher’s exact test was used instead. Linear correlation between two continuous variables was explored using Pearson’s correlation (if the data were normally distributed) and Spearman’s correlation (for non-normally distributed data). Statistical significance was kept at p < 0.05.

## Results

A total of 226 low-risk term pregnant women were randomized into two equal groups: the intervention group (n = 113), who underwent serial sweeping and stretching, and the control group (n = 113), who did not. Data was collected over 18 months, from July 2023 to December 2024. At baseline, the two groups were comparable in demographic and obstetric characteristics, confirming successful randomization. The mean age was 25.73 years in the intervention group and 25.50 years in the control group (p = 0.712). The mean BMI was also similar between groups (22.59 kg/m^2^ vs. 22.58 kg/m^2^; p = 0.910).

Parity distribution (p = 0.584), mean Bishop’s score (4.01 vs. 4.12; p = 0.691), and mean cervical length (3.60 cm vs. 3.56 cm; p = 0.443) did not differ significantly. At the second visit, cervical parameters showed significant improvement in the intervention group compared with controls (Table 1). The intervention group demonstrated higher mean Bishop’s scores (6.58 vs. 4.63; p < 0.001) and shorter mean cervical lengths (3.01 cm vs. 3.44 cm; p < 0.001). A greater proportion of participants in the intervention group (n = 25, 22.1%) returned for follow-up within one week compared to the control group (n = 9, 8.0%) (p = 0.003).

**Table 1:** Comparison of labour characteristics and timings at second visit between the two groups n: number of study participants in each group; p < 0.05 was considered statistically significant; TVS: transvaginal sonography

Characteristic	Intervention Group (n = 113)	Control Group (n = 113)	Statistical Test	p-value
1. Bishops score (second visit), mean	6.58	4.63	Wilcoxon-Mann-Whitney U (w = 9796.0)	<0.001
2. Cervical length on TVS (second visit, cm), mean	3.01	3.44	Wilcoxon-Mann-Whitney U (w = 3170.5)	<0.001
3. Time to next visit, n (%)				
(a) <1 week	25 (22.1)	9 (8.0)	Chi-Squared (χ^2 ^= 8.863)	0.003
(b) 1 week	88 (77.9)	104 (92.0)		
4. Period of gestation at onset of labour (weeks), mean	40.48	40.56	Wilcoxon-Mann-Whitney U (w = 5648)	0.128
5. Overall onset of labour (hours), mean	21.28	24.29	Wilcoxon-Mann-Whitney U (w = 4556.0)	<0.001
(a) Primigravida	23.51	25.20	Wilcoxon-Mann-Whitney U (w = 797.500)	0.074
(b) Multigravida	20.32	23.69	Wilcoxon-Mann-Whitney U (w = 1526.500)	<0.001
6. Overall duration of labour (hours), mean	13.57	19.08	Wilcoxon-Mann-Whitney U (w = 3392.0)	<0.001
(a) Primigravida	16.33	22.01	t-test (t = -4.661)	<0.001
(b) Multigravida	12.00	17.15	Wilcoxon-Mann-Whitney U (w = 1277.0)	<0.001

The mean onset of labour was significantly shorter in the intervention group (21.28 hours) than in the control group (24.29 hours) (p < 0.001), although the gestational age at onset of labour was comparable (p = 0.128). Among multigravidas, the onset was notably earlier (20.32 vs. 23.69 hours; p < 0.001), while the difference among primigravidas did not reach statistical significance (p = 0.074). The mean duration of labour was also significantly shorter in the intervention group (13.57 hours) compared with controls (19.08 hours) (p < 0.001), with similar trends among both primigravida and multigravida women.

The rate of labour induction was significantly lower in the intervention group (n = 33, 29.2%) than in controls (n = 78, 69.0%) (p < 0.001). This reduction was consistent across both primigravidas (n = 19, 46.3% vs. n = 42, 93.3%; p < 0.001) and multigravidas (n = 14, 19.4% vs. n = 36, 52.9%; p < 0.001) (Table 2). Similarly, the need for labour augmentation was lower in the intervention group (n = 94, 83.2%) compared with the control group (n = 111, 98.2%) (p < 0.001). Among multigravidas, augmentation was required less frequently in the intervention group (n = 54, 75.0%) than in controls (n = 66, 97.1%) (p < 0.001), while among primigravidas, the difference was not significant (p = 0.477). Participants who underwent augmentation or induction in both groups experienced longer labour durations (p < 0.001 for augmentation in the intervention group, p < 0.001 for induction in both groups, and p = 0.021 for augmentation in the control group).

**Table 2:** Comparison of labour interventions between the two groups n: number of participants in each group; p < 0.05 was considered statistically significant

Intervention	Intervention Group (n = 113)	Control Group (n = 113)	Statistical Test	p-value
1. Induction of labour, n (%)				
(a) Yes	33 (29.2)	78 (69.0)	Chi-squared (χ^2 ^= 35.852)	<0.001
(b) No	80 (70.8)	35 (31.0)		
2. Induction of labour by parity, n (%)				
(a) Primigravida: Yes	19 (46.3)	42 (93.3)	Chi-squared (χ^2^ = 22.976)	<0.001
(b) Multigravida: Yes	14 (19.4)	36 (52.9)	Chi-squared (χ^2^ = 17.091)	<0.001
3. Augmentation of labour, n (%)				
(a) Yes	94 (83.2)	111 (98.2)	Chi-squared (χ^2^ = 15.172)	<0.001
(b) No	19 (16.8)	2 (1.8)		
4. Augmentation of labour by parity, n (%)				
(a) Primigravida: Yes	40 (97.6)	45 (100.0)	Fisher's exact (χ^2^ = 1.110)	0.477
(b) Multigravida: Yes	54 (75.0)	66 (97.1)	Chi-squared (χ^2^ = 13.897)	<0.001

The rate of vaginal delivery (including instrumental deliveries) was significantly higher in the intervention group compared with controls (p < 0.001), whereas the caesarean section rate was higher in the control group (Table 3). Neonatal outcomes were comparable between groups, with no significant differences in neonatal intensive care unit (NICU) admissions (p = 0.622), one-minute APGAR scores (p = 0.744), and five-minute APGAR scores (p = 0.158).

**Table 3:** Mode of delivery and neonatal outcomes n: number of participants in each group; p < 0.05 was considered statistically significant; LSCS: lower segment cesarean section; APGAR: Appearance, Pulse, Grimace, Activity, Respiration; NICU: neonatal intensive care unit

Characteristic	Intervention Group (n = 113)	Control Group (n = 113)	Statistical Test	p-value
1. Mode of delivery, n (%)				
(a) Vaginal	107 (94.7)	90 (79.6)	Fisher's exact (χ^2^ = 14.523)	<0.001
(b) LSCS	6 (5.3)	23 (20.4)		
2. Postpartum hemorrhage, n (%)				
(a) No	113 (100.0)	113 (100.0)	Chi-squared	N/A
3. APGAR 1 minute, mean	7.44	7.40	Wilcoxon-Mann-Whitney U (w = 6244.0)	0.744
4. APGAR 5 minutes, mean	9.00	8.96	Wilcoxon-Mann-Whitney U (w = 6497.0)	0.158
5. NICU admission, n (%)				
(a) Yes	1 (0.9)	3 (2.7)	Fisher's exact (χ^2^ = 1.018)	0.622
(b) No	112 (99.1)	110 (97.3)		

**Figure 2:**
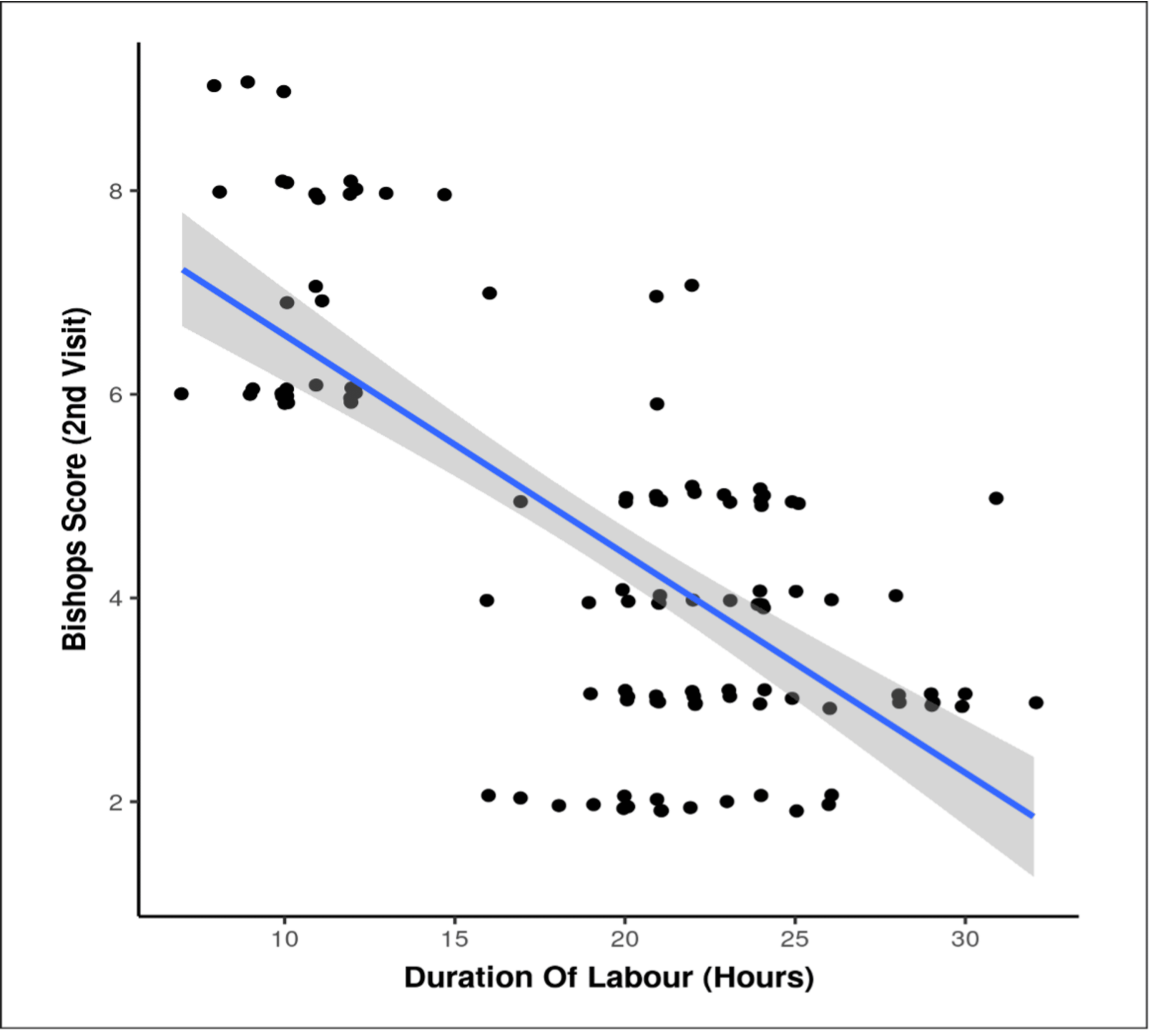
Distribution of Bishop’s score at the second visit in the intervention versus control group X-axis: duration of labour (hours); Y-axis: Bishop’s score at second visit (mean) Note: Duration of labour (hours) versus Bishop’s score at second visit showed a significant negative correlation (Spearman correlation coefficient = -0.6, p < 0.001).

**Figure 3:**
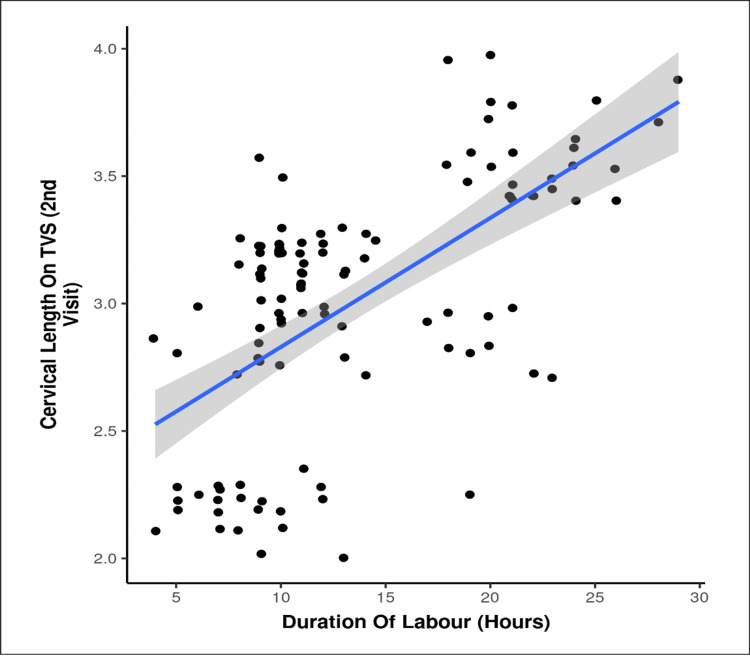
Distribution of cervical length on transvaginal sonography (TVS) at the second visit in the intervention versus control group X-axis: duration of labour (hours); Y-axis: cervical length on TVS at second visit (cm) Note: Duration of labour (hours) versus cervical length on TVS at second visit showed a significant negative correlation (Spearman correlation coefficient = -0.6, p < 0.001).

Correlation analyses revealed significant relationships between cervical status and labour progression. In both groups, higher Bishop’s scores were negatively correlated with the duration (intervention: ρ = -0.7, p < 0.001; control: ρ = -0.6, p < 0.001)) and onset of labour (intervention: ρ = -0.6, p < 0.001; control: ρ = -0.4, p < 0.001) (Figure 2), indicating that greater cervical favourability was associated with shorter labour. Conversely, cervical length on TVS showed a positive correlation with both labour duration (intervention: ρ = 0.6, p < 0.001; control: ρ = 0.4, p < 0.001) and onset of labour (intervention: ρ = 0.6, p < 0.001; control: ρ = 0.6, p < 0.001), suggesting that shorter cervical lengths predicted earlier and faster labour progression (Figures 2-3).

In summary, the serial sweeping and stretching of membranes significantly improved cervical ripening, shortened both the onset and duration of labour, and reduced the need for pharmacological induction and augmentation. The intervention also resulted in higher vaginal delivery rates without compromising maternal or neonatal safety outcomes. Correlation analyses reinforced that a favourable cervical status was strongly associated with faster and more efficient labour progression.

## Discussion

This study evaluated the effectiveness of serial membrane sweeping and stretching for labour induction among low-risk term pregnancies. Baseline variables, including age, BMI, parity, initial Bishop’s score, cervical length, and other demographic and obstetric parameters, were comparable between groups, confirming successful randomization. This methodological robustness was in agreement with the findings of Batham and Anjum [12] and Saichandran et al. [13].

Cervical ripening and Bishop’s score

The intervention group demonstrated significant improvement in cervical ripening at the second visit, as reflected by shorter mean cervical length and higher mean Bishop’s scores [14]. These results were consistent with the findings of Tan et al. [15], who reported that cervical shortening predicted a greater likelihood of vaginal delivery (p = 0.034) and reduced the need for induction and cesarean delivery. Similarly, de Miranda et al. [11] observed that membrane sweeping promoted a pre-labor state characterized by cervical softening and effacement. In contrast, Jeewantha et al. [16], in a primigravida-only cohort, reported no significant improvement, emphasizing that parity and study population composition influenced outcomes. Among 240 primigravidas, no difference was observed in mean Bishop’s score between participants who underwent one versus two sweep-stretch procedures by 40+5 weeks.

Onset and progression of labor

In the present study, serial sweeping and stretching significantly increased the rate of spontaneous labour (70.8% vs. 31.0%) and reduced the need for medical induction (29.2% vs. 69.0%), although the overall gestational age at the onset of labour did not differ significantly, likely due to standardized induction protocols. These findings were supported by multiple studies, including those by Hassan [17], Jeewantha et al. [16], Finucane et al. [18], de Miranda et al. [11], Zamzami and Al Senania [19], Pirzada et al. [20], and Ali et al. [3], which demonstrated increased spontaneous labor rates following membrane sweeping. Saichandran et al. [13] reported no significant difference in the need for oxytocin augmentation, artificial rupture of membranes, or spontaneous progression. Similarly, a meta-analysis by Wong et al. [10] and studies by Batham and Anjum [12] and Shams and Nasreen [21] yielded variable results, likely due to methodological and population heterogeneity. Conversely, Ugwu et al. [22] documented an earlier onset of labor in the intervention group, while Shams and Nasreen [21] found this effect more pronounced among multiparous women, suggesting that parity and baseline cervical favorability modulated the response to sweeping.

Induction rates and duration of labor

Both primigravida and multigravida participants in the intervention group exhibited significantly lower induction rates. Furthermore, the mean duration of labor was shorter in the sweep-and-stretch group (13.57 hours) compared with the control group (19.08 hours), consistent with the findings of Yildirim et al. [23]. In contrast, Batham and Anjum [12] found no such difference, possibly due to fewer interventions or smaller sample sizes. Notably, even among those requiring induction, participants in the intervention group experienced shorter labor durations, indicating enhanced cervical readiness and uterine efficiency. In accordance with Saichandran et al. [13], oxytocin augmentation was required less frequently in the intervention group (83.2% vs. 98.2%). Shams and Nasreen [21] observed a similar trend, particularly in multiparous women, likely attributable to more favorable cervical conditions. Importantly, participants who required augmentation in the intervention group still had shorter labor durations compared to controls, underscoring the facilitative effect of membrane sweeping on labor progression. These findings contrasted with those of Batham and Anjum [12] and Jeewantha et al. [16], possibly due to variations in intervention frequency and study design.

Mode of delivery and safety profile

Consistent with Saichandran et al. [13], the present study demonstrated a significant increase in vaginal delivery rates in the intervention group. Moreover, findings from Zamzami et al. [19], Pirzada et al. [20], and Njoku et al. [24] reaffirmed that serial sweep-and-stretch procedures were safe, as evidenced by comparable maternal and neonatal outcomes, including postpartum hemorrhage rates. No adverse events attributable to the intervention were observed, further supporting its safety in low-risk term pregnancies.

The present study, conducted in a single tertiary centre, limits the representation of the sample and findings due to potential variation in patient demographics and facility resources. Therefore, a multicentric study conducted over a longer duration could offer a more comprehensive perspective by encompassing a diverse range of patient populations and clinical settings. This approach would strengthen the reliability of conclusion drawn from the research.

## Conclusions

Serial membrane sweeping and stretching is an effective, safe, and practical method to enhance cervical ripening and promote the spontaneous onset of labor in low-risk term pregnancies. In this randomized study, the intervention significantly improved Bishop’s scores, shortened cervical length, increased spontaneous labor rates, and reduced the need for induction. Even among women requiring medical induction or augmentation, the procedure facilitated shorter labor durations, indicating improved cervical favorability and uterine efficiency. Importantly, maternal and neonatal outcomes remained comparable between groups, confirming a favorable safety profile.

Given its simplicity, low cost, and non-pharmacologic nature, serial membrane sweeping represents a valuable strategy for labor preparation in appropriately selected patients. However, as this study was conducted at a single tertiary center, multicentric research with larger and more diverse populations is recommended to strengthen the generalizability of these findings and refine clinical recommendations.
